# Annotations

**Published:** 1899-08-05

**Authors:** 


### Annotations.

### Medical Defence and Medical Aid.

An important step has been taken by the Medical Defence Union in regard to medical aid associations and other bodies by which touting for patients is carried on. It has been resolved in future to require intending candidates for membership of the Union, in addition to signing the usual application form, to sign a declaration that they do not at present hold any office in connection with any medical aid or other institution in which systematic canvassing is practised as a means of procuring patients, and that, in the event of being elected, they will not accept any offices whatsoever in, or be otherwise associated with any society, association, provident dispensary, or medical institution where canvassing for the purpose of procuring patients is practised: and will not engage in any practice either as principal, partner, or assistant in which canvassing for the purpose of procuring patients is adopted. This is a very strong resolution, and its effect will be far-reaching. In these days of blackmailing it is becoming every year more necessary to belong to a defence union, and if men who connect themselves with shady practices find that they cannot get the protection which such unions give, a strong impulse will be given to continue in the paths of rectitude. This action will also tend to restrict the choice of the medical aid associations more than ever to a class of men who will never do them any good or bring them any credit, and by so doing will cut at the very root of their success, for it must be remembered that it is on the credit and repute of their medical officers that such associations trade.

### The Doctor as a Carrier of Disease.

An American contemporary raises again the old cry that the doctor is too often a means of disseminating disease. We do not think that there is much in it. It would be quite unfair to argue from the elaborate methods adopted by the surgeon to secure asepsis that the physician who does not take all these precautions is a source of danger. The cases are not analogous, for when the surgeon in old days carried infection from operation to operation he did so by means of what was practically an inoculation; he actually inserted it into a raw wound. Still, that there is some risk of carrying infection in one's clothes, if one is careless and allows them to come in contact with an infectious patient, must be admitted. If cabs and omnibuses can be sources of infection why not doctor's coats? So far as the hands are concerned ordinary antiseptics and measures of cleanliness ought to be sufficient to prevent them ever becoming vehicles of disease. But the clothes are more difficult of control, and if dust is to be regarded as infectious it must sometimes be impossible to avoid transporting infectious particles from house to house. Moreover there are other ways in which a person who is much exposed to certain infections may carry them about. It is pretty well recognised that certain pathogenic microbes, notably those of diphtheria, may make for themselves a home on the mucous membrane of the air passages, as, for example, in the nose and pharynx, where they may continue to grow without producing any sign of disease. Whether this is due to a gradually-acquired immunity on the part of their host or to a loss of virulence on their own part it may be difficult to determine, but it seems clear that, however slight their virulence may be in regard to the person who carries them, they are capable of very quickly developing virulence if implanted in someone else, and this is a mode by which infection may possibly be spread by medical men, and still more, probably, by nurses, and one agains which it seem almost impossible to guard. Indeed, we should look upon the nurse or the mother, who spends long hours in the sick room, as much more likely to become a carrier of infection than the doctor, who merely looks in now and again. Still, the risk is there. On the whole, it would seem that the danger of carrying infection from case to case lies principally, although not entirely, in the possibility of carrying dust from house to house. The hands can always be kept clean, the nails can always be kept well trimmed, and probably few doctors get so soaked in infection that their mucous membranes become culture media for its germs; but clothes are a difficulty. Clearly, a medical practitioner ought to be a very spruce and well-brushed individual. A frowsy doctor may become a danger. But then he ought to be an anachronism.

### Real Physicians.

The absurd confusion which exists as to the meaning of the word physician seems interminable. The Hunter case having, for the present at any rate, decided that an L.S.A. cannot legally call himself a physician, the question now crops up, Who may use the title? and more particularly, Does the licence to practise medicine, surgery, and midwifery, and to dispense drugs, which is granted by the Royal College of Physicians of London, make a man a physician? This matter is brought to the front by the position taken up of late by certain people who maintain that not only the licentiates of the Apothecaries' Society, but medical graduates of the Universities, are "usurping" the title. Now, if it were to be maintained that none were physicians except the Fellows and members of the College we could understand it. There might then be something to argue about. But what have the licentiates to do with the question at all? What does it matter whether a man gets his licence to practise medicine, surgery, and midwifery from Pall Mall or from Blackfriars? In each case he receives it from a body empowered by an Act of Parliament to give it, but in neither case does he become a member of the body by which it is granted. The licentiates of the College of Physicians of London are, in fact, mere outsiders who have received licences to carry on certain lines of practice—licences "which," as is specially said in the bye-laws, "do not extend to make the licentiates members of the corporation." The licence is, in fact, merely a permit allowing the holder "to practice medicine, surgery, and midwifery so long as he shall continue to obey the statutes," &c. In fact, such a complete outsider is the holder of this document that he has not to go through any form of admission when it is granted. He signs a "promise" to obey the regulations, he pays a fee, he carries off the paper in his pocket, and then he has nothing more to do with the College, unless he misbehaves himself, when without more ado his licence can be revoked. Yet these good people are pretending to claim that they forsooth are "*real* physicians," and that medical graduates are mere usurpers if they take the title, even though, as a fact, they may be physicians to great hospitals. The contention does but serve to show the ridiculous position in which the whole question of medical qualifications and medical titles at present stands.

